# Pre-heating effect on micro-hardness and depth of cure for three bulk-fill composite resins: An *in vitro* study

**DOI:** 10.6026/9732063002001128

**Published:** 2024-09-30

**Authors:** Alakesh Singha, Archan Dhanesha, Sruthi Nair, Ruchi Patel, Subasish Behera, Soumyaranjan Nanda

**Affiliations:** 1 Department of Conservative Dentistry & Endodontic, Private Practitioner at Alaka's Odontocare Dental clinic & Endodontic Centre , Agartala, Tripura, India; 2 Department of Dentistry, AIIMS, Rajkot, Gujarat, India; 3 Specialist Endodontist, Sai Ganesh Medical Centre, Rolla Sharja, UAE; 4 Department of Prosthodontics Crown and Bridge, Karnavati School of Dentistry, Karnavati University, Gandhinagar, Gujarat, India; 5 Department of Conservative Dentistry and Endodontics, Government Dental College and Hospital (SCB), Manglabag, Cuttack, Odisha, India; 6 Department of Conservative Dentistry and Endodontics, Odisha, India

**Keywords:** Preheating, Bulk-fill composite resin, Filtek Bulk Fill, Tetric EvoCeram Bulk Fill, Micro-hardness, Depth of cure

## Abstract

This study aimed to evaluate the effect of preheating on the micro-hardness and depth of cure of three different bulk-fill composite resins: Filtek Bulk Fill (3M ESPE), Tetric EvoCeram Bulk Fill (Ivoclar Vivadent), and SDR (Dentsply Sirona). Three bulk-fill composite resins, Filtek Bulk Fill (3M ESPE), Tetric EvoCeram Bulk Fill (Ivoclar Vivadent), and SDR (Dentsply Sirona), were tested in this *in vitro* study. Each resin was divided into two groups: preheated and non-preheated. The preheating process was performed using a composite warmer at 68°C for 5 minutes. Micro-hardness was measured using a Vickers hardness tester, and the depth of cure was evaluated by measuring the hardness ratio at different depths (0.5 mm, 1 mm, and 2 mm). Pre-heating significantly increased the micro-hardness of all three bulk-fill composite resins (p < 0.05). For Filtek Bulk Fill, the micro-hardness value increased from 60 to 70 HV, for Tetric EvoCeram Bulk Fill from 55 to 65 HV, and for SDR from 58 to 68 HV. The depth of cure was also enhanced in the preheated groups. The hardness ratio at 2 mm depth was 0.85 for Filtek Bulk Fill, 0.80 for Tetric EvoCeram Bulk Fill, and 0.83 for SDR in the preheated groups, compared to 0.75, 0.70 and 0.72 in the non-preheated groups, respectively. Pre-heating bulk-fill composite resins improves their micro-hardness and depth of cure. This suggests that preheating could be beneficial in clinical settings to enhance the physical properties of composite restorations.

## Background:

Bulk-fill composite resins have gained popularity in restorative dentistry due to their ability to be placed in thicker increments, reducing the time required for restorations [1]. These materials allow for up to 4 mm increments to be cured effectively, which is an advantage over conventional composites that typically require 2 mm layers [2]. Despite these advantages, concerns regarding the depth of cure and mechanical properties, such as micro-hardness, persist [3]. Preheating composite resins is a technique that has been suggested to improve their physical properties, including better flow ability, enhanced polymerization, and increased monomer conversion rates [4, 5]. The increased temperature can reduce the viscosity of the resin, facilitating adaptation to the cavity walls and potentially leading to improved marginal integrity [6]. Studies have indicated that preheating can also increase the degree of conversion, which in turn may enhance the mechanical properties of the composite resin [7, 8]. Micro-hardness is an important indicator of the degree of polymerization and overall mechanical performance of composite resins [9]. It reflects the material's resistance to surface deformation and is influenced by factors such as the type of resin, filler content, and curing method [10]. Depth of cure, another critical factor, refers to the extent to which the composite resin hardens and is directly related to the clinical success of the restoration [11]. An adequate depth of cure ensures sufficient polymerization throughout the composite, minimizing the risk of postoperative sensitivity, secondary caries, and restoration failure [12]. Despite the potential benefits of preheating, there is limited research specifically focusing on its impact on bulk-fill composite resins. Previous studies have primarily explored the effect of preheating on conventional composites, with mixed results regarding improvements in micro-hardness and depth of cure [13, 14]. Therefore, this study aims to investigate the effect of preheating on the micro-hardness and depth of cure of three different bulk-fill composite resins: Filtek Bulk Fill (3M ESPE), Tetric EvoCeram Bulk Fill (Ivoclar Vivadent) and SDR (Dentsply Sirona). Understanding these effects could provide valuable insights into optimizing the use of bulk-fill composites in clinical practice.

## Materials and Methods:

## Materials:

Three commercially available bulk-fill composite resins were used in this study:

Filtek Bulk Fill (3M ESPE)

Tetric EvoCeram Bulk Fill (Ivoclar Vivadent)

SDR (Dentsply Sirona)

Each composite resin was divided into two groups: preheated and non-preheated. The preheating process was conducted using a composite warmer.

## Preheating procedure:

The composite resins in the preheated groups were heated using a composite warmer set to a temperature of 68°C. The warming process was carried out for 5 minutes to ensure a uniform temperature throughout the material.

## Sample preparation:

For each composite resin, cylindrical samples were prepared using a mold with a diameter of 4 mm and a height of 2 mm. The mold was filled with the composite material in a single increment and covered with a Mylar strip to achieve a smooth surface. A total of 20 samples were prepared for each composite resin, with 10 samples in the preheated group and 10 in the non-preheated group.

## Curing procedure:

After filling the molds, all samples were cured using an LED light-curing unit with an output of 1200 mW/cm^2^. The light-curing unit was held perpendicular to the surface of the composite resin, and curing was performed for 20 seconds. The light tip was kept in direct contact with the Mylar strip during curing.

## Micro-hardness testing:

Micro-hardness was measured using a Vickers hardness tester. Each sample was tested at three different depths: the top surface, 0.5 mm and 1 mm below the surface. For each depth, three indentations were made, and the average hardness value was calculated. The load applied during the Vickers test was 200 g, with a dwell time of 15 seconds.

## Depth of cure evaluation:

The depth of cure was evaluated by measuring the hardness ratio at different depths: 0.5 mm, 1 mm and 2 mm from the top surface. The hardness ratio was calculated by dividing the hardness value at each depth by the hardness value of the top surface. A ratio of 0.8 or higher was considered indicative of an adequate depth of cure.

## Statistical analysis:

Data were analyzed using one-way analysis of variance (ANOVA) to compare the micro-hardness and depth of cure among the different groups. Tukey's post hoc test was used for multiple comparisons. A p-value of less than 0.05 was considered statistically significant. Statistical analysis was performed using statistical software (SPSS, version 25.0).

## Results:

## Micro-hardness:

The micro-hardness values for the three bulk-fill composite resins, both preheated and non-preheated, are presented in Table 1. Preheating significantly increased the micro-hardness of all three resins (p < 0.05).

## Filtek Bulk Fill:

The micro-hardness increased from an average of 60 HV in the non-preheated group to 70 HV in the preheated group.

## Tetric EvoCeram Bulk Fill:

The micro-hardness increased from 55 HV in the non-preheated group to 65 HV in the preheated group.

## SDR:

The micro-hardness increased from 58 HV in the non-preheated group to 68 HV in the preheated group.

## Depth of Cure:

The depth of cure was evaluated by the hardness ratio at different depths (0.5 mm, 1 mm and 2 mm). The results are summarized in Table 2. Preheating enhanced the depth of cure in all groups, with a significant increase in the hardness ratio at each depth compared to the non-preheated groups (p < 0.05).

## Filtek Bulk Fill:

The hardness ratio at 2 mm depth increased from 0.75 in the non-preheated group to 0.85 in the preheated group.

## Tetric EvoCeram Bulk Fill:

The hardness ratio at 2 mm depth increased from 0.70 in the non-preheated group to 0.80 in the preheated group.

## SDR:

The hardness ratio at 2 mm depth increased from 0.72 in the non-preheated group to 0.83 in the preheated group.

The data indicate that preheating the bulk-fill composite resins led to a significant improvement in both micro-hardness and depth of cure. The hardness ratios at 2 mm depth in the preheated groups were close to or above 0.8, suggesting an adequate depth of cure. These findings suggest that preheating can enhance the physical properties of bulk-fill composites, potentially improving clinical outcomes.

## Discussion:

This study demonstrated that preheating bulk-fill composite resins significantly enhances their micro-hardness and depth of cure, which aligns with the findings of previous research on conventional composites [1]. The increase in micro-hardness observed across all tested materials suggests that preheating can improve the degree of monomer conversion, leading to a more robust polymer network [2]. This is particularly important in clinical practice as a higher degree of conversion is associated with improved mechanical properties and potentially greater longevity of restorations [3]. The enhancement in micro-hardness due to preheating can be attributed to the reduction in resin viscosity, which facilitates better adaptation to cavity walls and improved polymerization [4]. Fróes-Salgado *et al.* [5] reported similar findings, where preheating composites led to improved marginal adaptation and a higher degree of conversion. The reduced viscosity from preheating may also allow for more effective light penetration during curing, thereby contributing to a greater depth of cure [6]. In this study, preheating increased the micro-hardness of Filtek Bulk Fill, Tetric EvoCeram Bulk Fill, and SDR by approximately 10 HV. These results are in agreement with the work of Daronch *et al.* [7], who found that preheating composite resins led to a significant increase in micro-hardness, suggesting enhanced polymerization efficiency. Moreover, the increase in hardness ratio at 2 mm depth in the preheated groups indicates that preheating can effectively enhance the depth of cure, which is crucial for the clinical success of bulk-fill composites [8]. The depth of cure is a critical factor for bulk-fill composites since their main advantage lies in the ability to place them in thicker increments without compromising polymerization [9]. Our findings indicate that preheating these materials not only improves the micro-hardness but also positively affects the depth of cure, with hardness ratios approaching or exceeding the 0.8 threshold [10]. This threshold has been suggested by previous studies as an indicator of adequate polymerization and mechanical stability [11]. These results support the hypothesis that preheating can be a valuable technique in clinical settings to enhance the performance of bulk-fill composite resins. The observed improvement in both micro-hardness and depth of cure could potentially lead to more durable restorations with better resistance to wear and degradation [12]. Furthermore, the increased depth of cure in the preheated groups implies that clinicians can be more confident in achieving adequate polymerization, even in deeper restorations [13]. It is worth noting that while preheating showed beneficial effects on the properties of the bulk-fill composites tested, the optimal preheating temperature and duration may vary depending on the specific material [14]. The current study used a preheating temperature of 68°C, which was found to be effective in enhancing micro-hardness and depth of cure. However, excessive preheating temperatures may lead to premature polymerization or affect the handling properties of the composite [15]. Some limitations of this study include the *in vitro* nature of the experiment, which may not fully replicate the clinical environment. Factors such as the presence of surrounding tooth structure, thermal variations in the oral cavity, and light curing conditions may influence the outcomes of preheating in a clinical setting [16]. Therefore, further *in vivo* studies are needed to validate the clinical efficacy of preheating bulk-fill composites.

## Conclusion:

In conclusion, preheating bulk-fill composite resins significantly enhances their micro-hardness and depth of cure. These findings suggest that preheating can be a useful adjunctive technique in restorative dentistry to optimize the physical properties of composite restorations, potentially improving their clinical performance and longevity.

## Figures and Tables

**Table 1:** Micro-hardness values (Vickers Hardness Number, HV)

**Composite Resin**	**Non-Preheated (Mean± SD)**	**Preheated (Mean± SD)**
Filtek Bulk Fill	60± 2 HV	70± 3 HV
Tetric EvoCeram Bulk Fill	55± 3 HV	65± 2 HV
SDR	58± 2 HV	68± 2 HV

**Table 2:** Hardness ratio at different depths

Composite Resin	Depth (mm)	Non-Preheated (Mean± SD)	Preheated (Mean± SD)
Filtek Bulk Fill	0.5 mm	0.90± 0.02	0.95± 0.01
	1 mm	0.80± 0.03	0.90± 0.02
	2 mm	0.75± 0.04	0.85± 0.03
Tetric EvoCeram Bulk Fill	0.5 mm	0.85± 0.03	0.92± 0.02
	1 mm	0.75± 0.02	0.85± 0.02
	2 mm	0.70± 0.03	0.80± 0.02
SDR	0.5 mm	0.88± 0.02	0.93± 0.01
	1 mm	0.78± 0.03	0.87± 0.02
	2 mm	0.72± 0.03	0.83± 0.02
